# Memory Strategy Use, Cognition, and Metacognition Among People Aging with HIV

**DOI:** 10.1007/s10461-026-05076-8

**Published:** 2026-02-24

**Authors:** Jasmine K. Vickers, Jeremy D. Delgadillo, Shakaye R. Haase, Alexis R. Long, Philemon Domoyeri, David E. Vance, Pariya L. Fazeli

**Affiliations:** 1 School of Nursing, University of Alabama at Birmingham, Birmingham, AL USA; 2 Department of Psychology, University of Alabama at Birmingham, Birmingham, AL USA; 3 School of Nursing, University of Alabama at Birmingham, 1720 2nd Ave. South, Magnolia Office Park - Plaza Building Suite #227, Birmingham, AL 35294-1210 USA

**Keywords:** HIV, Internal-external control, Memory disorders, Memory strategies, Metacognition

## Abstract

Living with the HIV virus is a chronic health condition with life expectancies among treated people similar to seronegative individuals. However, older people living with HIV (PLWH) are at higher risk for comorbidities, including cognitive impairment, than their seronegative counterparts. Use of memory strategies has been shown to enhance memory performance among older PLWH, yet few studies have examined factors that drive memory strategy use as well as how impairments in metacognition may hinder the initiation of memory strategies. To address this gap, associations between cognitive function, locus of control and memory strategy use among PLWH with and without memory impairment were examined, followed by the association between metacognition and memory strategy use among PLWH. A secondary analysis was performed using cross-sectional data of 174 PLWH aged 40+ years. Spearman’s correlation was used to determine associations between memory strategy use (total, internal, and external), objective cognitive performance (seven domains and global), and locus of control (internal and external), and informed follow-up linear regression models. ANOVA was used to assess differences in memory strategy use between PLWH across metacognition groups (i.e., concordance between subjective and objective memory functioning yielding four groups: agree both normal, agree both impaired, over-estimators, under-estimators). Among PLWH with memory impairment, higher use of total memory strategies was associated with higher SES, White race, and higher external locus of control (powerful others subscale). Higher use of internal memory strategies was associated with higher SES, White race, older age, and higher external locus of control (powerful others and chance subscales). Higher external strategy use was associated with better executive function. Among PLWH without impairment, higher use of total memory strategies was associated with younger age. Higher use of internal memory strategies was associated with poorer global cognition, executive, and learning function. Higher external strategy use was associated with female sex, younger age, and higher SES. Higher internal locus of control was correlated with better function in all cognitive domains among PLWH without memory impairment. Among all PLWH, those who underestimated their cognitive ability used the most memory strategies, while those who overestimated their abilities used the least memory strategies. Those who correctly estimated their cognitive abilities as being normal or impaired fell in the middle with correct estimators of impaired cognition using the second most memory strategies. As PLWH get older, education around the use of effective memory strategies as well as social support to reinforce memory strategy use may be key to promoting improved confidence in cognitive abilities and quality-of-life as cognition declines. PLWH who overestimate their cognitive abilities and people from low socioeconomic status backgrounds may be less likely to use memory strategies and may benefit from being targeted for memory strategy education.

## Introduction

Advances in the treatment for HIV have transformed it from a terminal illness into a manageable chronic condition with life expectancies approaching that of people living without HIV (PLWoH) [1]. A longer life expectancy is contributing to increasing numbers of middle-aged and older adults living with HIV in the United States. Over 50% of people living with HIV (PLWH) are estimated to be 50 years old and older, and this rate is expected to increase to 70% by 2030 [2]. PLWH are at higher risk of cognitive impairment than PLWoH [3, 4]. Estimates suggest that compared to PLWoH, PLWH have up to a sevenfold risk of mild cognitive impairment, a precursor to Alzheimer’s disease [5].

Compensatory memory strategy use has been posited as an effective neurorehabilitation approach to enhance cognition and everyday function among older PLWH [6]. For older PLWH, memory strategy use has been associated with better working memory, executive function, verbal performance [6], learning, and recall [7] domains of cognition. Memory strategy use for coping with working memory deficits have been shown to be particularly helpful for older adults [8]. Memory strategy use may decrease the load on working memory, allowing for better encoding and more cognitive power for higher level functions [6]. However, the lack of memory strategy use in PLWH has been found to be correlated with vulnerabilities such as low educational attainment and belonging to a racial minority group [7]. Among PLWoH, external memory strategy use (using strategies external to oneself such as writing things down on a calendar or setting an alarm) is associated with older age, while internal strategy use (using strategies that involves the use of resources within oneself such as creating an acronym or mnemonic strategy for a list of items to be remembered) is associated with higher executive function [9]. Lin and colleagues found that older PLWoH with memory impairment were more likely to use external memory strategies that involved the assistance of another person (such as asking a family member to remind them of a task later) [10].

Metacognition, one’s awareness of cognitive abilities, may also impact memory strategy use. Estimates suggest that 43% of PLWH with cognitive impairment have metacognitive deficits [11] which can impact functional abilities [12]. A lack of awareness of deficient cognitive abilities may decrease motivation for and use of memory strategies that could enhance function [11, 13]. Locus of control (LOC), a person’s perceived control of their life, is another factor that may impact cognitive performance via complex relationships with metacognition, cognition, and memory strategy use [14, 15]. Internal versus external memory strategies and LOC may have differing associations. As defined by Rotter [16], external LOC is the perception that major causes of events are controlled by others, a powerful entity (e.g., god, fate, powerful others), or by chance. Internal LOC is the perception that major causes of events are controlled by oneself through their behavior. In a sample of older PLWoH, those with mild cognitive impairment had higher external LOC compared to cognitively intact older adults. Additionally, higher external LOC was associated with more overreporting of cognitive symptoms [14]. Regarding LOC and memory strategy use, another study among PLWoH found internal LOC and internal memory strategy use declines with age and this is thought to be related to age-related declines in working and episodic memory [15].

The relationship between metacognition and memory strategy use among PLWH with and without memory impairment is not well understood and can inform neurorehabilitative efforts for older PLWH. This study utilized comprehensive measures of memory strategies, metacognition, cognitive function, and LOC to assess these relationship dynamics. We first examined associations between cognitive function and LOC with memory strategy use among PLWH with and without memory impairment. We then examined the association between metacognition and memory strategy use among PLWH.

## Methods

### Study Design

This study was a secondary analysis of the Successful Aging with HIV/AIDS study data [17, 18], which was a cross-sectional study examining predictors of successful aging among PLWH. This study enrolled 174 PLWH who were recruited from an HIV/AIDS specialty clinic associated with an academic medical center in the southeastern United States (Deep South). Potential participants were contacted by a phone call for screening and eligibility. Participants were included if they were 40 years of age or older and a patient at an HIV clinic. Participants were excluded if they had comorbidities that affected cognition (i.e., Stroke, Alzheimer’s disease, Schizophrenia, a head injury that resulted in a loss of consciousness), major sensory deficits, or were receiving chemotherapy or radiation. This study was approved by the institution’s review board for ethical research practices (IRB-160601006). Participants received an explanation of the study and provided written informed consent before completing a one-time assessment with the measures below.

### Measures

#### Memory Strategies

The Multifactorial Memory Questionnaire (MMQ) is a 57-item self-report survey that assesses three domains: satisfaction with memory (18 items), memory mistakes (20 items), and memory strategy use (19 items) [19]. Higher scores on the MMQ translates to more satisfaction with memory, less memory mistakes, and more frequent use of memory strategies. The satisfaction subscale measures how individuals feel about their memory using items such as “my memory is worse than most other people my age”. Responses are scored from 0 to 4, with reverse coding for several items, using a Likert scale with five options (strongly agree, agree, undecided, disagree, strongly disagree). The memory mistakes subscale measures the frequency of twenty memory mistakes in the past two weeks (e.g., item #1 “forget to pay a bill on time”). Responses are scored from 0 to 4 with five response options (all the time, often, sometimes, rarely, never). The memory strategy use subscale measures the frequency of using nineteen memory strategies and is further broken down into internal (e.g., item #19 “mentally retrace your steps to remember something…”) and external (e.g., item #9 “use a routine to remember important things…”) strategy use to which participants rate frequency on a scale ranging from 0 = never to 4 = all the time [19]. The satisfaction, mistakes, and memory strategy subscales demonstrated high internal consistencies (α = 0.95, α = 0.94, and α = 0.90 respectively) in our study. Memory strategy use scores (external, internal, and total) are used in analyses while the satisfaction with memory and memory mistakes subscales are used for calculating the metacognition variable described below.

#### Neurocognitive Function

A gold-standard neurocognitive battery was used to assess cognitive function and included verbal, executive functioning, speed of information processing, learning, recall, working memory, and motor cognitive domains along with a global score [20]. Test scores for each cognitive domain were converted into demographically corrected T-scores, which were based on normative corrections [21]. T-scores were used in analyses and reported in the results for all cognitive domains. To determine memory impairment, T-scores were converted to clinical ratings [22] with higher scores indicating greater impairment. Memory impairment constituted a clinical rating of five or greater in the domain of learning or recall, which were assessed with two tests: the Hopkins Verbal Learning Test-Revised and the Brief Visuospatial Memory Test-Revised [23, 24].

#### Metacognition

Metacognition, one’s awareness of cognitive abilities, was calculated using the memory impairment variable (described above) and a subjective memory functioning variable (using the MMQ memory satisfaction and mistakes subscales) as has been described [7]. A threshold for subjective memory functioning was determined by assigning an impaired subjective memory function designation if the total sum of the MMQ memory satisfaction and mistakes scores fell below 49.5% of all participant scores. Then four metacognitive groups were calculated from the objective and subjective memory impairment dichotomous variables and include (1) agree: both normal, (2) agree: both impaired, (3) under-estimators, and (4) over-estimators. The *agree both normal* group had no subjective or objective impairment and the *agree both impaired* group had objective and subjective impairment. *Under-estimators* had no objective impairment but endorsed subjective impairment. *Over-estimators* had objective impairment but did not endorse subjective impairment.

#### Locus of Control

The Personality in Intellectual Aging Contexts Inventory Control Scales Short Form is a 6-item instrument used to measure cognitive and intellect-specific LOC beliefs in older adults [25, 26]. The instrument contains three domains that assess internal (items #1 and 2), chance (items #3 and #4), and powerful others (items #5 and #6) LOC. Internal LOC refers to the belief that cognitive and intellectual abilities are under one’s own control (e.g., item #2 “It’s up to me to keep my mental faculties from deteriorating”). Chance and powerful others subscales are both considered an external LOC meaning abilities are not within one’s own control. Chance refers to the belief that cognitive and intellectual abilities are controlled by external forces (e.g., item #4 “I have little control over my mental state”). Powerful others refers to the belief that help from other people is needed for the individual to successfully accomplish cognitive tasks (e.g., item #5 “I can’t figure out sales prices of items unless someone helps me”). Each question contains 6 Likert-scale response options that range from *strongly agree* (1) to *strongly disagree* (6). Internal LOC items are reverse coded so that higher scores indicate higher internal LOC. Lower scores on the chance and powerful others subscales indicate a higher external LOC. Therefore, total scores range from 6 to 36 with higher scores representing higher internal LOC beliefs for cognition and intellect. The scale demonstrated good reliability in our study sample (Cronbach alpha α = 0.70).

#### Demographics

Demographics included age at baseline assessment, sex (male or female), work status, race, and viral load. Socioeconomic status (SES) was calculated using sample-based z-scores of education, income, and verbal IQ measures, as is described [7], with higher scores reflecting a higher SES.

### Analysis

IBM SPSS Statistics version 30.0.0.0 (IBM Corp 2024) was used for analysis. Spearman’s correlation coefficients were used to test associations between memory strategy use, cognition, and LOC between and within memory impaired and non-impaired groups. Significant correlations were modeled using linear regressions to assess associations between memory strategy use and/or LOC on cognitive function adjusting for conceptually relevant covariates. ANOVA was used to assess the differences of memory strategy means across the four metacognitive groups while adjusting for conceptually relevant covariates. Two-sided tests with a cut off value of 0.05 denoting statistical significance were used.

## Results

The sample for this study included 174 PLWH with a mean age of 51 years ±7.0 (40–73). Participants were mostly male (62%), non-White (86% African American, Native American, or other race), very low income (90% with $30,000 household income or less), on disability (54%), had a low education level (78% some college or less), and an HIV viral load of less than 20 copies per milliliter of blood (66%). Less than half of participants (41%) had memory impairment. PLWH with memory impairment were more likely to be White than the non-memory impaired group (*p*=0.011). Global cognition and all seven cognitive domains were significantly better in the memory unimpaired group. Gender, age, SES, memory strategy use, and LOC did not differ between those with and without memory impairment. We also examined whether individual memory strategies differed by memory impairment groups and found that memory strategy number seven (i.e., organizing information for better memory) was the only memory strategy that had a statistically significant difference (*p*=0.028) such that the unimpaired memory group reported using this strategy more (see Table 1). The most frequently reported memory strategies in the sample (those reported to be performed on average “sometimes” or more frequently) were items five (“write things on a calendar”), nine (“use a routine to remember important things), ten (“make a list”), and twelve (“put something in a prominent place to remind you to do something”).

**Table 1:** Participant characteristics by memory impairment (*n* = 174)

Variable		Memory Impairment (*n* = 71)	No Memory Impairment (*n* = 103)	*p*-value
Age M±SD		50.5 ± 6.5	51.8 ± 7.4	0.235
Sex n(%)	Male	41(38.0%)	67(62.0%)	0.329
	Female	30(45.5%)	36(54.5%)	
Race n(%)	White	16(64.0%)	9(36.0%)	0.011*
	Non-White	55(36.9%)	94(63.0%)	
SES M±SD		−0.84 ± 1.7	−0.49 ± 2.2	0.263
Memory Strategies M±SD	Total	32.2 ± 14.2	35.2 ± 13.4	0.152
	Internal	14.9 ± 8.6	16.7 ± 8.3	0.163
	External	17.3 ± 6.9	18.5 ± 6.9	0.246
Locus of Control M±SD	Total	27.9 ± 6.0	28.7 ± 5.8	0.417
	Internal	9.8 ± 2.5	9.8 ± 2.2	0.786
	Chance	8.8 ± 2.6	9.1 ± 2.5	0.338
	Powerful Others	9.4 ± 2.8	9.7 ± 2.7	0.539
Cognitive Function M±SD	Global	40.8 ± 4.7	48.0 ± 5.1	< 0.001*
	Verbal	42.6 ± 6.9	48.5 ± 7.3	< 0.001*
	Executive	42.7 ± 8.9	49.2 ± 8.2	< 0.001*
	Speed of Processing	44.5 ± 8.1	50.3 ± 7.7	< 0.001*
	Learning	36.9 ± 5.3	47.4 ± 5.3	< 0.001*
	Recall	37.3 ± 5.9	48.5 ± 5.8	< 0.001*
	Working Memory	42.0 ± 7.2	48.0 ± 9.3	< 0.001*
	Motor	36.7 ± 9.3	42.8 ± 8.1	< 0.001*
MMQ MS#1 Timer/Alarm M±SD		1.6 ± 1.4	1.7 ± 1.4	0.531
MMQ MS#2 Reminder from Someone M±SD		1.8 ± 1.3	1.8 ± 1.1	0.872
MMQ MS#3 Rhyme M±SD		0.62 ± 0.88	0.79 ± 0.99	0.255
MMQ MS#4 Image M±SD		1.3 ± 1.2	1.2 ± 1.1	0.908
MMQ MS#5 Calendar M±SD		2.9 ± 1.3	3.0 ± 1.1	0.662
MMQ MS#6 Alphabet M±SD		0.85 ± 1.2	1.1 ± 1.4	0.178
MMQ MS#7 Organize Information M±SD		1.1 ± 1.3	1.6 ± 1.4	0.028*
MMQ MS#8 Repeat Out Loud M±SD		1.8 ± 1.2	2.2 ± 1.3	0.073
MMQ MS#9 Routine M±SD		2.4 ± 1.2	2.5 ± 1.3	0.593
MMQ MS#10 List M±SD		2.4 ± 1.2	2.6 ± 1.3	0.398
MMQ MS#11 Focus on Details M±SD		1.9 ± 1.2	2.1 ± 1.1	0.195
MMQ MS#12 Prominent Placement M±SD		2.3 ± 1.1	2.5 ± 1.1	0.131
MMQ MS#13 Repeat at Longer Intervals M±SD		1.7 ± 1.2	1.7 ± 1.2	0.898
MMQ MS#14 Create a Story M±SD		1.1 ± 1.1	0.88 ± 1.0	0.283
MMQ MS#15 Write Down in Notebook M±SD		2.1 ± 1.3	2.3 ± 1.3	0.385
MMQ MS#16 Acronym M±SD		0.62 ± 0.96	0.81 ± 1.1	0.238
MMQ MS#17 Concentrate M±SD		1.7 ± 1.3	1.9 ± 1.3	0.363
MMQ MS#18 Write a Note M±SD		1.9 ± 1.3	2.2 ± 1.4	0.168
MMQ MS#19 Retrace Steps M±SD		2.2 ± 1.2	2.4 ± 1.1	0.223

*SES* social economic status, *MMQ MSS* Multifactorial Memory Questionnaire Memory Strategy Subscale

*p *≤ 0.05*

See Table 2 (Memory Impairment, *n* = 71) and 3 (No Memory Impairment, *n* = 103) for memory strategy, LOC, and cognition correlations within each memory group. Among PLWH with memory impairment, higher use of memory strategies (total) was associated with higher SES, White race, and higher external LOC (powerful others subscale). Higher use of internal memory strategies was associated with higher SES, White race, older age, and higher external LOC (powerful others and chance subscales). The only significant correlate of cognition was the association between higher external memory strategy use and higher executive function. There were no associations between LOC and cognition among the PLWH with memory impairment.

**Table 2:** Spearman’s correlation matrix for memory strategies in participants with memory impairment (*n* = 71)

Variable		1	2	3	4	5	6	7
Memory Strategies	1. Total	1						
	2. Internal	0.923**	1					
	3. External	0.851**	0.610**	1				
Locus of Control	4. Total	− 0.196	− 0.202	− 0.134	1			
	5. Internal	0.034	0.032	0.025	0.686**	1		
	6. Chance	− 0.229	− 0.270*	− 0.090	0.788**	0.311**	1	
	7. Powerful Others	− 0.267*	− 0.255*	− 0.213	0.809**	0.382**	0.533**	1
Age		0.231	0.263*	0.099	0.289*	0.304*	0.133	0.210
Sex (male = 0, female = 1)		− 0.116	− 0.219	0.010	− 0.239*	− 0.370**	− 0.068	− 0.117
Race (Non-white = 0, White = 1)		0.263*	0.234*	0.195	0.176	0.388**	0.035	0.037
SES		0.267*	0.265*	0.194	0.272*	0.357**	0.121	0.155
Cognitive Function	Global	0.092	0.027	0.153	0.148	0.084	0.136	0.126
	Verbal	0.108	0.054	0.148	0.046	0.007	0.124	0.004
	Executive	0.163	0.079	0.251*	0.072	0.085	0.103	0.004
	Speed of Processing	− 0.013	− 0.059	0.057	0.220	0.130	0.184	0.181
	Learning	0.096	0.057	0.125	− 0.038	− 0.030	0.007	− 0.063
	Recall	0.129	0.051	0.194	− 0.119	− 0.111	− 0.043	− 0.166
	Working Memory	0.063	0.078	0.061	0.152	0.126	0.120	0.101
	Motor	− 0.033	− 0.027	− 0.066	0.056	0.113	− 0.049	0.069

*SES *social economic status

*p < 0.05 **p < 0.001

Among PLWH without impairment, higher use of memory strategies (total) was associated with younger age. Higher use of internal memory strategies was associated with poorer global, executive, and learning function. Higher external strategy use was associated with female sex, younger age, and higher SES. Higher internal LOC, as indicated by the total LOC variable, was correlated with better function in all cognitive domains, and there were several domain-specific correlations with the internal and external LOC subscales as well (Table 3).

**Table 3:** Spearman’s correlation matrix for memory strategies in participants without memory impairment (*n* = 103)

Variable		1	2	3	4	5	6	7
Memory Strategies	1. Total	1						
	2. Internal	0.898**	1					
	3. External	0.837**	0.537**	1				
Locus of Control	4. Total	− 0.004	− 0.071	0.110	1			
	5. Internal	− 0.030	− 0.094	0.104	0.693**	1		
	6. Chance	− 0.057	− 0.090	0.024	0.798**	0.347**	1	
	7. Powerful Others	0.047	− 0.009	0.316	0.818**	0.376**	0.539**	1
Age		− 0.239*	− 0.168	− 0.231*	− 0.011	0.044	− 0.091	− 0.027
Sex (male = 0, female = 1)		0.167	− 0.001	0.303**	− 0.123	− 0.157	− 0.031	− 0.034
Race (Non-white = 0, White = 1)		0.141	0.138	0.126	0.201*	0.191	0.026	0.204*
SES		0.179	0.072	0.252*	0.585**	0.401**	0.351**	0.550**
Cognitive Function	Global	− 0.157	− 0.234*	− 0.045	0.431**	0.250*	0.264**	0.483**
	Verbal	− 0.148	− 0.176	− 0.089	0.339**	0.191	0.269**	0.303**
	Executive	− 0.095	− 0.198*	0.049	0.287**	0.228*	0.169	0.278**
	Speed of Processing	− 0.110	− 0.171	− 0.052	0.358**	0.256**	0.158	0.411**
	Learning	− 0.165	− 0.200*	− 0.087	0.383**	0.216*	0.235*	0.432**
	Recall	− 0.175	− 0.192	− 0.109	0.224*	0.094	0.086	0.324**
	Working Memory	0.069	0.928	0.124	0.305**	0.043	0.221*	0.388**
	Motor	− 0.029	− 0.054	0.000	0.206*	0.215*	0.143	0.164

*SES* social economic status

*p < 0.05 **p < 0.001

Based on results from the correlations, we then conducted the following data-driven regressions using cognitive function as dependent variables (DV) and memory strategies or LOC as independent variables (IV): model 1 (memory impairment group: executive function [DV] and external memory strategies [IV]), model 2 (memory unimpaired group: global cognitive function [DV], internal memory strategies [IV], and powerful others LOC [IV]), model 3 (memory unimpaired group: executive function [DV], internal memory strategies [IV], and total LOC [IV]), and model 4 (memory unimpaired: learning [DV], internal memory strategies [IV], and powerful others LOC [IV]). All models included conceptually relevant covariates: age, sex, race, and SES. No memory strategy or LOC independent variables were associated with the cognition dependent variables after adjusting for demographic covariates except for lower internal memory strategies and lower powerful other’s LOC association with better learning among memory unimpaired individuals (Table 4).

**Table 4:** Regression model associations for cognitive domains

	β	SE	t	*p*
Memory Impairment Group - Model 1 Executive Functioning *n* = 93				
External Memory Strategies	0.179	0.129	1.386	0.169
Age	0.062	0.156	0.398	0.692
Sex (male = 0, female = 1)	0.380	1.89	0.201	0.841
Race (Non-white = 0, White = 1)	0.922	2.62	0.353	0.725
SES	− 0.248	0.580	− 0.428	0.669
Memory Unimpaired Group- Model 2 Global Functioning *n* = 81				
Internal Memory Strategies	− 0.059	0.058	−1.01	0.317
Powerful Others Locus of Control	0.343	0.192	1.79	0.078
Age	− 0.024	− 0.042	− 0.406	0.686
Sex (male = 0, female = 1)	− 0.600	0.981	− 0.611	0.543
Race (Non-white = 0, White = 1)	−1.05	− 0.076	− 0.673	0.503
SES	0.830	0.230	3.60	< 0.001
Memory Unimpaired Group- Model 3 Executive Functioning *n* = 81				
Internal Memory Strategies	− 0.117	0.110	−1.07	0.290
Total Locus of Control	0.132	0.175	0.750	0.456
Age	− 0.119	0.112	−1.06	0.292
Sex (male = 0, female = 1)	−1.41	1.86	− 0.757	0.452
Race (Non-white = 0, White = 1)	1.06	2.93	0.361	0.719
SES	0.873	0.455	1.92	0.059
Memory Unimpaired Group- Model 4 Learning *n* = 81				
Internal Memory Strategies	− 0.147	0.068	−2.16	0.034
Powerful Others Locus of Control	0.618	0.224	2.75	0.007
Age	− 0.035	0.070	− 0.496	0.621
Sex (male = 0, female = 1)	−1.57	1.15	−1.37	0.175
Race (Non-white = 0, White = 1)	−1.71	1.83	− 0.933	0.354
SES	0.370	0.270	1.37	0.175

All models adjusted for the covariates age, sex, race, SES and were entered simultaneously

**Fig. 1:**
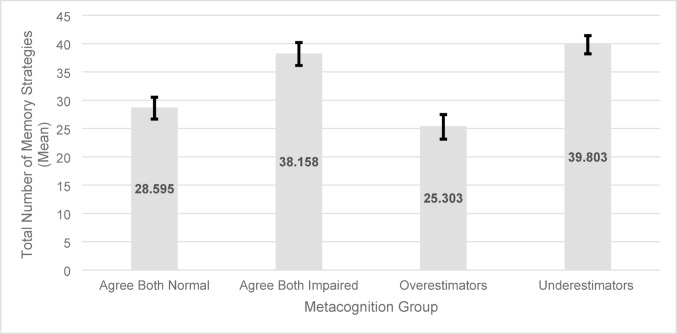
The means of total number of memory strategies used by metacognition group

In the full PLWH sample, the total number of memory strategies had a significant association with metacognition F(3,170) = 13.77, *p*<0.001. Those with metacognitive deficits who underestimated their cognitive ability used the most memory strategies (39.8), while those who overestimated their abilities used the fewest memory strategies (25.3) (Fig. 1). Those who correctly estimated their cognitive abilities as being normal (28.6) or abnormal (38.2) fell in the middle, with correct estimators of abnormal cognition using the second most memory strategies. When conceptually relevant covariates were added to the model (age, sex, race, and SES), the association between metacognition and memory strategies remained F(3,170) = 13.89, *p*<0.001.

## Discussion

We aimed to test the associations between memory strategy use, cognition, LOC, and metacognition among a sample of middle-aged and older PLWH from the Deep South. We first compared PLWH with and without memory impairment on study variables and found as expected the memory impaired PLWH had poorer cognitive performance in all domains as well as global functioning. These groups did not differ on LOC or memory strategy subscales; however, we found that PLWH without memory impairments used the memory strategy organizing information more frequently. This may suggest that memory strategies that require more cognitive demand to perform may be used less by people with memory impairments, yet, despite this difference, both groups reported low frequency of this activity. The most frequently reported memory strategies that were reported as being performed on average “sometimes” or greater were: writing things on a calendar, using a routine to remember important things, and putting something in a prominent place to remind you to do something. These reflect more typical or obvious memory strategies, whereas more abstract strategies involving cognitive mnemonic strategies, such as using acronyms and mental images, were used less frequently. This may represent a future area for intervention among PLWH with memory impairment. Prior work has demonstrated improvements in memory among PLWH, with and without pre-existing memory impairment, using cognitive interventions such as the self-generation approach [27] and space retrieval practice [28]. A comprehensive cognitive intervention with similar approaches supplemented with practical memory strategies may be a feasible and low-cost approach to equipping PLWH, or increasing cognitive functioning of PLWH and memory impairment, with tools for cognitive success as they age.

Our findings revealed that among PLWH with memory impairment, higher use of memory strategies (total and internal) was associated with higher SES, White race, and a LOC for cognitive health more reliant on external sources, specifically the powerful others subscale. This speaks to more socially advantaged populations with memory impairment being more likely to use memory strategies while having perceptions of less control over their cognitive abilities. Using more memory strategies could lead to better adaptation and functioning with memory impairment. However, as these individuals were experiencing memory impairments, encouragement to use external strategies more frequently may be more advantageous for cognitive and everyday function. Guerrero-Sastoque and colleagues found that older adults are less likely to self-initiate memory and recall strategies to improve cognitive processing as compared to younger adults [29], and they may require external support with age-related or pathological changes in cognition.

Among PLWH without memory impairment, higher use of memory strategies (total) was associated with younger age. Higher external strategy use was associated with female sex, younger age, and higher SES. PLWH without memory impairment may benefit from memory strategy use to be able to withstand higher cognitive demands and to be equipped with tools in case cognition declines, since they are at higher risk for decline than PLWoH. There may be unique psychosocial barriers, such as embarrassment, that discourage the use of external memory strategies in certain subgroups (such as older PLWH, males, and PLWH from low SES backgrounds) that should be explored in future research. Since SES is frequently associated with memory strategy use in those with and without memory impairment, PLWH with low income or education may benefit from educational or memory strategy interventions to bolster their feelings of control in their cognition as they age.

Among PLWH with memory impairment, there was an association between higher external memory strategy use and higher executive function. Higher executive functioning may be required to strategize successfully to employ external memory strategies. This suggests that PLWH with memory impairment require self-efficacy building or education around the utility of external memory strategies for them to employ them regularly. Additionally, individuals living with or caring for PLWH with memory impairment may need to encourage the use of external strategies, as PLWH with lower executive function may forget or find external memory strategies too complicated. This association between higher external memory strategy use and higher executive function may also reflect that memory strategy use can improve executive functioning, however this cross-sectional study limits causal inference.

Among PLWH without memory impairment, higher use of internal memory strategies was associated with poor global, executive, and learning function. PLWH may intuitively deploy internal memory strategies in response to cognitive difficulties; however, teaching external memory strategies in addition to internal memory strategies may more effectively improve cognitive function as cognition declines. Future research is needed to test these relationships longitudinally. Among PLWH without memory impairment, a higher internal LOC was correlated with better function in all cognitive domains, and a stronger external LOC (chance and powerful others) was associated with poorer cognitive function in several domains. These findings may suggest that lower cognitive function decreases one’s sense of control in their cognitive abilities or that lower perceptions of internal control decrease cognitive performance. Either way, teaching memory strategies to PLWH and an external LOC may help them build more self-efficacy related to their cognitive abilities. Future research is needed to test these relationships.

Most of the independent associations of memory strategy use and LOC with cognition were not significant in covariate adjusted models. This suggests that factors such as SES may be more impactful on cognitive functioning, and they may obscure the independent associations of memory strategy and LOC on cognition in PLWH. The one significant regression showed that, after adjusting for covariates, lower use of internal memory strategies and lower powerful other’s LOC were associated with better learning among memory unimpaired PLWH. This suggests that among older PLWH without memory impairment, relying on more internal memory strategies is associated with poorer learning abilities. This could also mean that PLWH without memory impairment with better learning abilities are less likely to adopt internal strategy use, while those with worse learning abilities are more likely to adopt internal strategy use.

Those with metacognitive deficits that underestimated their cognitive ability used the most memory strategies, while those that overestimated their abilities used the least memory strategies. It may be that the people who overestimated their abilities felt like they were competent in their cognition and thus did not need to use memory strategies. PLWH who overestimate their cognitive abilities may be less likely to use memory strategies and may benefit from being targeted for memory strategy education, particularly given that they demonstrated objective memory impairment. Furthermore, while those PLWH who underestimated their cognitive ability used the most memory strategies and had no objective impairment, continuing to use these strategies may be a beneficial resource in the future as they age with a higher risk for cognitive impairment due to their HIV infection.

### Limitations

This study was a cross-sectional study. Therefore, casual and temporal inferences cannot be drawn from this study. We could not ascertain if LOC, memory strategy use, nor metacognition affect cognitive function or vice versa. Future studies are needed to test these relationships longitudinally. Although we were able to assess education, we could not ascertain educational quality, which may be highly variable considering educational disparities in the Deep South for older adults. Education has been shown to be related to memory strategy use; therefore, educational quality may provide important insights that may have been missed. This study focused on PLWH in the Deep South and therefore may not be generalizable to PLWoH, PLWH in other regions of the United States, or internationally.

### Strengths

This study is the first, to our knowledge, to examine memory strategy use and LOC in depth with internal and external measures and their association with cognition and metacognition among PLWH with and without cognitive impairment. This nuanced assessment provides evidence for PLWH who may benefit the most from memory strategy interventions.

## Conclusions

Lack of memory strategy use and self-control in cognitive abilities in PLWH has been found to be correlated with vulnerabilities such as low socioeconomic status and may need to be explicitly taught to more disadvantaged groups. When controlling for sociodemographic covariates, memory strategy use and LOC effects diminish, signaling the importance of sociodemographics in cognitive functioning in PLWH. These barriers may be overcome by explicitly teaching memory strategy use, particularly external memory strategy use and including the teaching of family or friends of PLWH for support.
